# Hepatocellular response to acute kidney injury in the critically ill: serum induces *CYP2D6* transcription

**DOI:** 10.1186/2197-425X-3-S1-A627

**Published:** 2015-10-01

**Authors:** K Lane, JJ Dixon, IAM MacPhee, BJ Philips, M Dockrell

**Affiliations:** Critical Care, St George's University Hospitals NHS Foundation Trust, London, United Kingdom; Critical Care, St George's University of London, London, United Kingdom; Renal Medicine, St George's University of London, London, United Kingdom; St George's University Hospitals NHS Foundation Trust, London, United Kingdom; South West Thames Institute for Renal Research, Surrey, United Kingdom

## Introduction

Cytochrome P450 2D6 (CYP2D6) is a clinically important CYP, metabolising approximately 25% common drugs. We investigated the clinical effect of acute kidney injury (AKI) on hepatic CYP2D6 metabolism in critically ill adults, using the probe drug tramadol (abstract 470). We found no effect of AKI but a strong *CYP2D6* genotype/phenotype influence on tramadol metabolism.

Rodent studies indicate no change or impaired CYP2D6 metabolism in chronic kidney disease and AKI (Refs [1–3]). No published human or animal data has examined CYP2D6 transcription, translation or activity in AKI. Previously we demonstrated no change in *CYP2D6* transcription when pooled serum from patients with end-stage kidney disease (ESKD) was applied to human HepG2 cells, known to express the functional CYP2D6*1 allele.

## Objectives

We aimed to determine whether a transcriptional change occurred in CYP2D6 expression when hepatocytes are exposed to serum from critically ill patients with and without AKI.

## Methods

As part of a clinical study of hepatic drug metabolism in AKI, serum from critically ill adult patients was stored at -80ºC. Serum from 16 patients with the severest AKI (KDIGO 3, not yet on renal replacement, highest fold-change in serum creatinine) was compared to that of 15 critically ill controls without AKI. HepG2 cells (human hepatoma cell line) were exposed to medium with 10% individual human serum in separate wells for 24 h, then lysed. *CYP2D6* gene expression was examined by real time reverse transcriptase quantitative PCR (q-rt-RT-PCR). Statistical analysis was performed using Biorad CFX 3.1 Software.

## Results

The patient demographics are shown.

Cells displayed no obvious morphological differences.

An increase in relative *CYP2D6* transcription occurred (1.14 vs 1.00, p = 0.037) when cells were exposed to serum from individual patients with AKI compared to those without.

## Conclusions

In contrast to the clinical study finding that CYP2D6 metabolism is not altered by AKI, a significant change in increase mRNA transcription occurred when sera were individually tested. The functionality of this transcript is uncertain and whether it translates into increased cellular CYP2D6 protein concentration in AKI remains unknown.

**Figure 1:**
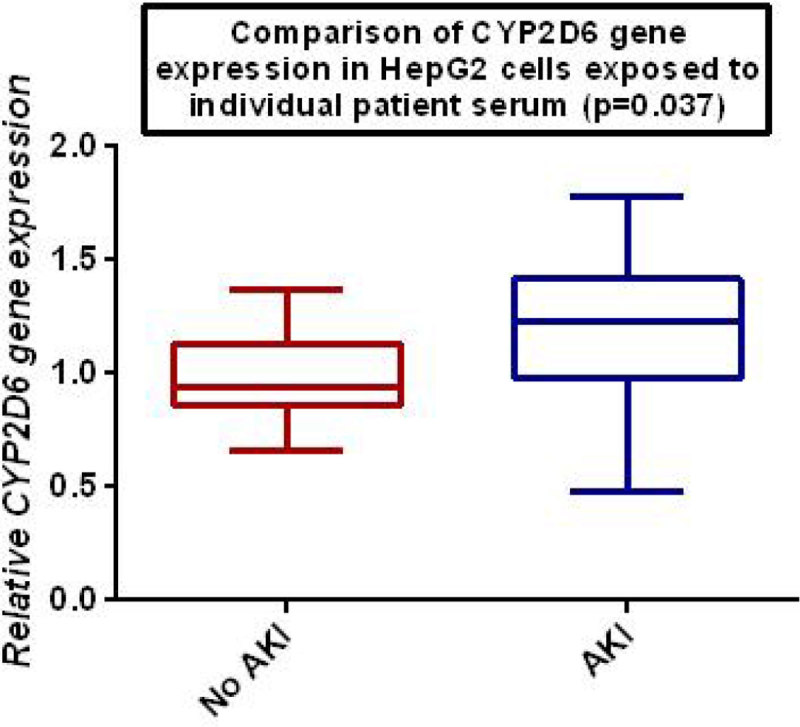
**CYP2D6 expression could not be evaluated by Western blotting, due to low expression of CYP2D6 in the cells (< 2% all CYP).**

**Table 1:** Patient Demographics for Critically Ill.

Value - Median [range]	Critically Ill, no AKI (n=15) (KDIGO 0)	Critically Ill, Severe AKI (n=16) (KDIGO 3)
Age (y)	77 (22-88)	58.5 (19-73)
Sex F:M	7:8	4:12
APACHE II score	20 (5-25)	25 (13-27)
SOFA score	7 (1-11)	7 (3-14)
Baseline Serum Creatinine (umol/L)	85 (58-112)	73 (46-114)
Fold change in Serum Creat from Baseline on study day	0.97 (0.5-1.16)	3.87 (3.1-10.1)
Serum Creatinine when studied (umol/L)	79 (41-131)	304 (157-595)
Cellular Confluence at Harvest (%)	86 (80-90)	85 (80-90)
[Tramadol] ((T4-T0) (ng/mL) [All genotypes included]	23.5 (14.3-39.8)	30.0 (11.7-41.4)

## Grant Acknowledgment

ESICM Basic Sciences Award, Springer Foundation and St George's Medical Charity.
